# Increased ROS and Persistent Pro-Inflammatory Responses in a Diabetic Wound Healing Model (db/db): Implications for Delayed Wound Healing

**DOI:** 10.3390/ijms26104884

**Published:** 2025-05-20

**Authors:** Hanan Elajaili, Bailey D. Lyttle, Caitlin V. Lewis, James R. Bardill, Nathan Dee, Sudipta Seal, Eva S. Nozik, Kenneth W. Liechty, Carlos Zgheib

**Affiliations:** 1 Cardiovascular Pulmonary Research Laboratories and Pediatric Critical Care Medicine, University of Colorado Anschutz Medical Campus, Aurora, CO 80045, USA; caitlin.v.lewis@cuanschutz.edu (C.V.L.); nathan.dee@cuanschutz.edu (N.D.); eva.nozik@cuanschutz.edu (E.S.N.); 2 Department of Surgery, University of Colorado, Anschutz Medical Campus, Aurora, CO 80045, USA; bailey.lyttle@cuanschutz.edu (B.D.L.); james.bardill@cuanschutz.edu (J.R.B.); 3 Biionix Cluster, Department of Internal Medicine, College of Medicine, University of Central Florida, Orlando, FL 32816, USA; sudipta.seal@ucf.edu; 4 Laboratory for Fetal and Regenerative Biology, Department of Surgery, College of Medicine, University of Arizona, Tucson, AZ 85721, USA; kliechty@arizona.edu

**Keywords:** diabetes, wound healing, db/db mouse model, chronic inflammation, dermal fibroblast, reactive oxygen species (ROS), EPR

## Abstract

Diabetes and its complications, including impaired wound healing, present a critical clinical challenge and burden for the U.S. healthcare system, with costs of over USD 13 billion annually. Hyperglycemia and chronic inflammation in diabetic wounds increase reactive oxygen species (ROS) production, inducing oxidative stress and perpetuating inflammation, which delays healing. This study investigates inflammation, oxidative stress, and the roles of cellular populations in a diabetic wound healing mouse model (db/db). Given that diabetes leads to persistent inflammation and impaired fibroblast function, we also examined how diabetes influences superoxide production in dermal fibroblasts. Blood, dermal fibroblasts, and wound tissue were collected from 12-week-old female diabetic (Db) and heterozygous (Hz) mice. Electron paramagnetic resonance (EPR) spectroscopy revealed higher superoxide levels in diabetic blood, dermal fibroblasts, and wounds compared to controls. In diabetic wounds, immunohistochemistry and flow cytometry showed increased leukocyte infiltration and reduced macrophage presence, with a higher proportion of pro-inflammatory Ly6C^hi^ macrophages. These results suggest that elevated superoxide production and persistent inflammation contribute to impaired fibroblast function and delayed wound healing in diabetes. By identifying the contributions of ROS and Ly6C^hi^ macrophages to oxidative stress and chronic inflammation, this study offers insights into therapeutic strategies. These findings highlight the importance of addressing systemic oxidative stress alongside localized inflammation to improve wound healing outcomes in diabetic patients and advance diabetic wound care strategies.

## 1. Introduction

Diabetes is a chronic metabolic disorder associated with multiple complications, including chronic wounds, which impose a significant burden on the U.S. healthcare system, costing over USD 13 billion annually. Impaired wound healing in diabetes is driven by persistent hyperglycemia, which increases reactive oxygen species (ROS) production. While ROS play a critical role in cell signaling, excessive levels contribute to oxidative stress, tissue damage, and chronic inflammation, creating a self-perpetuating cycle that hinders effective wound repair.

Diabetic wounds are characterized by prolonged inflammation, impaired growth factor signaling, abnormal angiogenesis, and defective collagen deposition, all of which contribute to delayed healing [1,2,3]. A key driver of this dysregulated repair process is the persistence of pro-inflammatory macrophages, which sustain chronic inflammation and exacerbate oxidative stress [3,4,5]. However, despite extensive research on diabetic wounds, the mechanisms by which diabetes alters oxidative stress and inflammation in intact skin remain less understood. Given that fibroblasts play a crucial role in both maintaining skin integrity and orchestrating wound repair, investigating oxidative stress in fibroblasts from non-wounded diabetic skin is critical to understanding how pre-existing cellular dysfunction contributes to impaired healing [6].

Understanding the cellular and molecular mechanisms underlying diabetic wound healing is essential to developing targeted therapies. In particular, the role of immune cells in sustaining chronic inflammation and their interaction with ROS provide valuable insight into potential therapeutic strategies [7,8,9,10,11]. By addressing these fundamental processes, innovative treatments may be developed to break the cycle of inflammation and oxidative damage, promoting effective wound resolution in diabetic patients. Given that diabetes is associated with systemic oxidative stress and persistent inflammation, we hypothesize that elevated superoxide production contributes to impaired fibroblast function and promotes a pro-inflammatory immune environment that delays wound healing in diabetic skin. Furthermore, we propose that this redox imbalance is evident not only in wounds but also in intact skin and circulating cells, indicating a systemic component to local healing defects. To test this hypothesis, we pursued the following specific aims: (1) to assess superoxide production in blood, dermal fibroblasts from intact skin, and wound tissue of diabetic (db/db) and control (heterozygous) mice using electron paramagnetic resonance (EPR) spectroscopy, to determine whether oxidative stress is systemic and pre-existing prior to injury, and (2) to characterize immune cell infiltration in diabetic wounds using immunohistochemistry and flow cytometry, including the identification of cell populations, to evaluate the inflammatory profile associated with impaired healing.

## 2. Results

### 2.1. Superoxide Levels Are Elevated in Diabetic Blood and Dermal Fibroblasts

**Figure 1:**
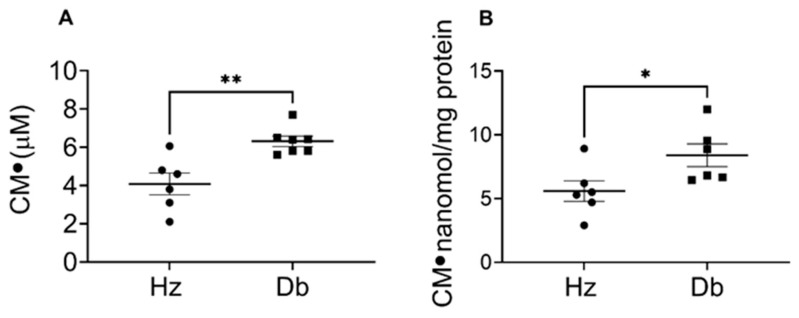
Increased superoxide levels in the blood and dermal fibroblasts of diabetic mice compared to heterozygous control mice. Whole blood was collected via cardiac puncture and fibroblasts were isolated from the skin of 12-week-old female control heterozygous (Hz) and diabetic (Db) mice. A total of 300 µL of the blood was treated with a CMH probe (0.25 mM) for 10 min at room temperature. Cultured fibroblasts were treated with a CMH probe (0.25 µM) and incubated for 50 min at 5% CO_2_ and 37 °C. A total of 50 µL of blood or cell suspension was loaded in a capillary tube and EPR measurements were conducted immediately. EPR measurements were conducted at room temperature and spectra were recorded using an X-band spectrometer EMXnano (Bruker). The amount of superoxide was determined based on the 1:1 reaction of CMH with superoxide to form CM^●^ and quantified by the SpinFit and SpinCount modules (Bruker). (**A**) Concentration of CM^●^ in blood. (**B**) Concentration of CM^●^ in fibroblasts normalized to protein concentration. Data were analyzed with Prism software version 10.4.2 using an unpaired *t*-test and are expressed as mean ± SEM; * *p* < 0.05, ** *p* < 0.01 (*n* = 6–7).

We first assessed oxidative stress by measuring superoxide production in both blood and dermal fibroblasts to determine the effects of diabetes. We used EPR spectroscopy and a hydroxylamine spin probe (CMH) to detect superoxide production. The CMH probe reacts with superoxide and generates a stable nitroxide radical that can be measured and quantified by EPR. Increased superoxide levels were observed in diabetic mice compared to heterozygous control mice (Figure 1A). Interestingly, although wounding did not further increase blood superoxide levels in either Db or Hz mice, the levels in Db mice remained consistently higher than in Hz mice (Figure S1A). Given that diabetes leads to persistent inflammation and impaired fibroblast function, we tested how diabetes influences superoxide production in dermal fibroblasts. Notably, we observed that diabetic dermal fibroblasts produce higher levels of superoxide compared to control mice (Figure 1B).

### 2.2. Superoxide Levels Are Elevated in Diabetic Wounds

**Figure 2:**
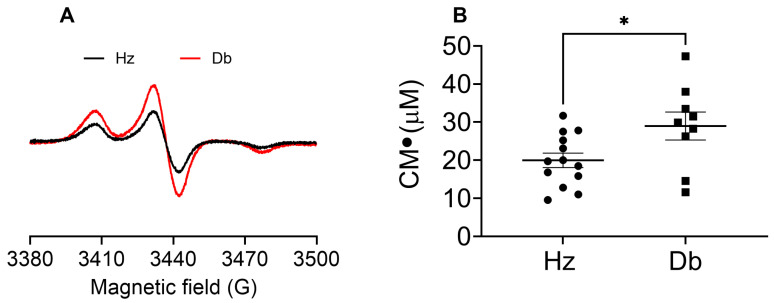
Increased superoxide levels in wounds from diabetic mice compared to heterozygous control mice. Wound tissue superoxide was measured 7 days after wounding. Skin from the wounds of heterozygous (Hz) and diabetic (Db) mice was homogenized in sucrose buffer, with a 1:6 ratio of tissue to buffer. The skin homogenate was treated with a CMH probe (0.25 mM) and incubated for 20 min at room temperature; then, 150 µL of the mixture was loaded into PTFE tubing and flash-frozen in liquid nitrogen. EPR measurements were conducted at 77 K and spectra were recorded using the X-band spectrometer EMXnano (Bruker). (**A**) Representative EPR spectra of the nitroxide CM^●^ in wounds from Db (red trace) and Hz (black trace). (**B**) CM^●^ concentration was obtained by double integration followed by SpinCount. Data were analyzed with Prism software version 10.4.2 using an unpaired *t*-test and are expressed as mean ± SEM; * *p* < 0.05 (*n* = 9–13).

We next evaluated oxidative stress in the wounds by measuring superoxide levels 7 days after wounding. Diabetic mice exhibited significantly higher superoxide levels in their wounds compared to control heterozygous mice (Figure 2A,B).

### 2.3. Diabetic Wounds Exhibit an Altered Immune Cell Repertoire Characterized by Increased Neutrophils, Decreased Total Macrophages, and an Increased Ratio of Pro-Inflammatory (Ly6C^hi^) Macrophages

**Figure 3:**
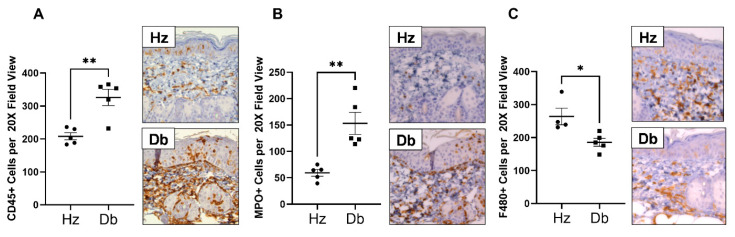
Increased total leukocyte and neutrophil infiltration but decreased macrophage infiltration in diabetic wounds compared to heterozygous controls. Diabetic and heterozygous wounds were assessed for inflammatory cell infiltration by immunohistochemistry, specifically staining for (**A**) CD45 (leukocytes), (**B**) myeloperoxidase (MPO, neutrophils), and (**C**) F480 (macrophages). High-power fields (HPFs, 200× magnification). Data were analyzed with Prism software version 10.4.2 using an unpaired *t*-test and are expressed as mean ± SEM; * *p* < 0.05, ** *p* < 0.01 (n = 4–5).

**Figure 4:**
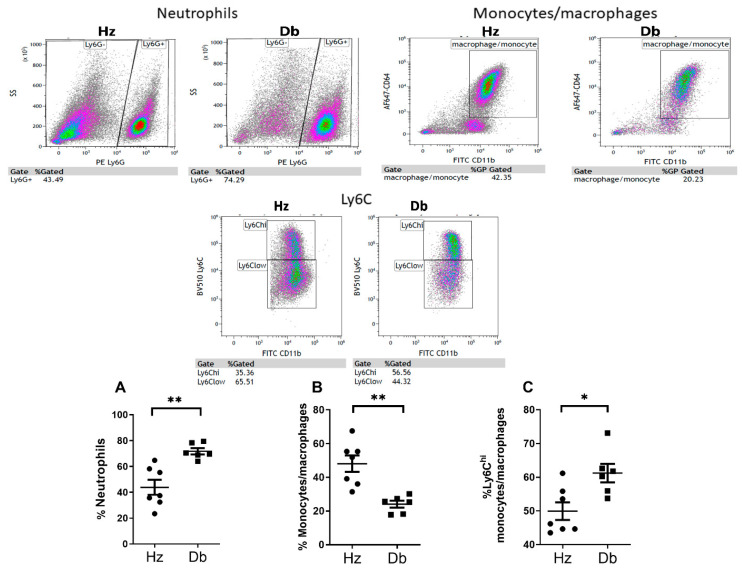
Fewer macrophages and a higher proportion of Ly6C^hi^ macrophages in diabetic wounds compared to heterozygous controls. The myeloid cell phenotype was assessed by flow cytometry in diabetic and heterozygous wounds: (**A**) neutrophil (CD64− Ly6G+), (**B**) monocyte/macrophage (CD64+ Ly6G-), and (**C**) monocyte/macrophage Ly6C^hi^ populations. Data were analyzed with Prism software version 10.4.2 using an unpaired *t*-test and are expressed as mean ± SEM; * *p* < 0.05, ** *p* < 0.01 (n = 6–7).

To assess the contribution of various cellular populations in diabetic wound healing, we evaluated the infiltration of inflammatory cells in the wounds 7 days after wounding. Diabetic and heterozygous wounds were assessed for inflammatory cell infiltration by immunohistochemistry, specifically staining for CD45 (leukocytes), myeloperoxidase (MPO, neutrophils), and F480 (macrophages). A significant increase in the total number of leukocytes was observed in diabetic wounds compared to heterozygous controls (Figure 3A). This included a dramatic increase in neutrophils (Figure 3B) and a decrease in macrophages (Figure 3C). This altered proportion of infiltrating inflammatory cells was further confirmed by flow cytometry, in which the percentage of neutrophils increased and the percentage of monocytes and macrophages decreased in diabetes (Figure 4A,B). Supporting a pro-inflammatory microenvironment in diabetic wounds, the proportion of monocytes and macrophages expressing the pro-inflammatory marker Ly6C was significantly increased (Figure 4C). Interestingly, this dysregulated inflammatory environment, prominent in diabetic wounds, was also reflected in the blood of diabetic mice. Blood analysis revealed altered leukocyte populations, including an increased proportion of neutrophils and monocytes expressing the pro-inflammatory marker Ly6C (Figure S1B–D), highlighting the systemic nature of inflammation in diabetes.

## 3. Discussion

Hyperglycemia in diabetes drives the overproduction of ROS, inducing oxidative stress, tissue damage, and impaired wound healing. In this study, we investigated the interplay between inflammation, oxidative stress, and cellular dynamics in a diabetic wound healing mouse model (db/db). Through analysis of ROS production and immune cell content, we identify fibroblasts as an important source of ROS in diabetic skin and reveal dysregulated inflammation associated with increased neutrophil and Ly6C^hi^ macrophage infiltration in diabetic wounds with delayed healing.

Our findings reveal systemic oxidative stress in diabetic mice, evidenced by significantly elevated superoxide levels in the blood, as measured by EPR spectroscopy. Notably, diabetic dermal fibroblasts exhibited increased superoxide production, reinforcing the notion that diabetes disrupts fibroblast function, a critical player in maintaining skin integrity and promoting repair [12,13]. While ROS are essential signaling molecules in normal wound healing, their excessive production in diabetic wounds perpetuates tissue damage and delays healing [14,15]. Indeed, superoxide levels were markedly higher in diabetic wound tissue compared to controls, pointing to sustained inflammation as a driving factor.

Furthermore, diabetic wounds demonstrated increased leukocyte and neutrophil infiltration alongside reduced macrophage presence, confirmed by flow cytometry. Importantly, the persistence of pro-inflammatory Ly6C^hi^ macrophages in diabetic wounds highlighted a disrupted transition to reparative Ly6C^lo^ macrophages, which would likely exacerbate chronic inflammation and delayed healing. This finding is consistent with previous reports [16,17,18,19,20], and our results demonstrate the presence of Ly6Chi cells in the db/db mouse model, supporting their association with diabetic inflammation.

Ly6C^hi^ macrophages are known to play a critical role in the early inflammatory phase of wound healing by producing high levels of pro-inflammatory cytokines (e.g., TNF-α, IL-1β, and IL-6), ROS, and matrix metalloproteinases (MMPs), which contribute to tissue degradation and delayed healing in diabetic wounds. In contrast, the Ly6Clo macrophages, which are typically reduced in this context, are associated with anti-inflammatory signaling and tissue remodeling, producing factors such as TGF-β, IL-10, and growth factors like VEGF, which promote fibroblast proliferation, collagen deposition, and extracellular matrix (ECM) remodeling [21].

In conclusion, our study underscores the dual role of oxidative stress and inflammatory dysregulation in diabetic wound pathology. By identifying elevated ROS and Ly6C^hi^ macrophages as central drivers of impaired repair, we provide a foundation for developing therapeutic strategies that target these pathways, aiming to disrupt the cycle of inflammation and oxidative damage for improved healing outcomes.

One limitation of this study is the challenge of isolating a sufficient number of macrophages from mouse wounds, and the concern that the isolation process itself can alter their activation state and ROS phenotype, potentially confounding the results. For these reasons, we opted to assess overall ROS levels in the wound tissue, capturing the broader oxidative stress environment. We used only female db/db mice for this study because these mice reliably develop severe, chronic, and non-healing wounds that closely mimic the impaired wound healing observed in diabetic patients. Female db/db mice are particularly well characterized for this model, with more consistent wound pathology and reduced aggressive behavior compared to males, which can influence wound outcomes. However, we recognize that this approach may limit the broader applicability of our findings, as sex differences can affect the wound healing process. Future studies including both male and female mice will be essential to fully understand the therapeutic potential of our intervention across sexes.

## 4. Materials and Methods

### Animal Model of Diabetic Wound Healing

Animal experiments were in compliance with guidelines outlined in the NIH Guide for the Care and Use of Laboratory Animals. All experimental protocols were reviewed and approved by the Institutional Animal Care and Use Committee at the University of Colorado Denver–Anschutz Medical Campus (License #84-R-0059).

Twelve-week-old female genetically diabetic (BKS.Cg-Dock7m+/+Leprdb/J(Db)) mice and heterozygous, non-diabetic (non-Db) age-matched female controls from the Jackson Laboratory (Bar Harbor, ME, USA) were used for this study. All mice were confirmed to have a blood glucose level greater than 300 mg/dL. All wounding procedures were performed under inhaled anesthesia with Isofluorane (3–5% induction and 1–3% maintenance), and mice received a single subcutaneous injection of Buprenorphine for post-procedural analgesia (Schering-Plough Animal HealthCorp, Kenilworth, NJ, USA). The posterior neck and back were shaved and depilated prior to wounding. The area was cleaned with an alcohol swab, and a single dorsal full-thickness wound was made with an 8 mm punch biopsy (Miltex, Inc., York, PA, USA).

## 5. Methods

### 5.1. Electron Paramagnetic Resonance (EPR) Spectroscopy

#### 5.1.1. Superoxide in the Blood

Blood was collected from 12-week-old female heterozygous and diabetic mice via cardiac puncture. Mice were deeply anesthetized using 1.5% isoflurane. Blood collection was performed using 21-gauge heparin-coated syringes. A volume of 300 µL of blood was treated with the cyclic hydroxylamine probe 1-Hydroxy-3-methoxycarbonyl-2,2,5,5-tetramethylpyrrolidine (CMH; 0.25 mM) in KHB buffer containing 100 µM diethylenetriaminepentaacetic acid (DTPA) and incubated for 10 min at room temperature. Following incubation, 50 µL of the treated blood was loaded into a capillary tube, and electron paramagnetic resonance (EPR) measurements were conducted immediately. EPR measurements were performed at room temperature using an X-band bench-top spectrometer (Bruker EMXnano, Billerica, MA, USA). The CMH probe reacts with superoxide and generates a stable nitroxide radical that can be measured and quantified by EPR. The concentration of nitroxide CM● generated from the reaction of CMH with superoxide was obtained by the SpinFit and SpinCount modules incorporated in EMXnano.

#### 5.1.2. Superoxide in Dermal Fibroblasts

Fibroblasts were isolated from the skin of 12-week-old female diabetic (Db) and heterozygous (Hz) control mice and cultured in Dulbecco’s Modified Eagle Medium (DMEM) supplemented with 10% fetal bovine serum and 1% antibiotic–antimycotic. Once the cells reached confluence, fibroblasts were seeded into 6-well plates and cultured until approximately 80% confluence. The cells were then treated in KHB buffer containing 100 µM diethylenetriaminepentaacetic acid (DTPA) and the CMH probe (0.25 mM). Following treatment, the cells were incubated for 50 min at 5% CO_2_ and 37 °C. Subsequently, 50 µL of the cell suspension was loaded into capillary tubes, and electron paramagnetic resonance (EPR) measurements were performed immediately. EPR measurements were performed using an X-band EMXnano spectrometer at room temperature. The concentration of CM● was obtained by the SpinFit and SpinCount modules and normalized to protein concentration.

#### 5.1.3. Superoxide in Wounds

Superoxide levels in wound tissue were measured 7 days post-wounding. Skin from the wound areas was homogenized in sucrose buffer at a tissue-to-buffer ratio of 1:6. The skin homogenate was then mixed with the CMH probe (0.25 mM) in KHB buffer containing 100 µM diethylenetriaminepentaacetic acid (DTPA) and incubated for 20 min at room temperature. Subsequently, 150 µL of the mixture was loaded into PTFE tubing and flash-frozen in liquid nitrogen. EPR measurements were conducted in the X-band at 77 K. The CM● concentration was determined by double integration followed by quantification using the SpinCount module.

#### 5.1.4. Immunohistochemistry

Diabetic and heterozygous wounds were assessed for inflammatory cell infiltration by immunohistochemistry. After full wound closure, the area of scar was excised and half the wound was fixed in 10% formalin, with the remaining wound used for biochemical analysis. After 24 h of formalin fixation, wounds were placed in 70% ethanol solution prior to paraffin embedding. Paraffin-embedded tissue was sectioned at 5 mm and then stained with Masson’s trichrome. Ten random high-power fields (HPFs, 200× magnification) of the trichrome-stained slides were imaged along the healed wound edge. Using an automated algorithm in NIS Elements–Advanced Research imaging software NIS-Ar 5.2. Slides used for immunohistochemistry were deparaffinized and placed in a citrate buffer (pH 6.0). The heat-induced epitope was retrieved with the Decloaker (Biocare Medical, Pacheco, CA, USA) and stained (Leica’s Bond Rx, Wetzlar, Germany) for CD45 (leukocytes), myeloperoxidase (MPO, neutrophils), and F480 (macrophages).

#### 5.1.5. Flow Cytometry

Wounds were collected in Hank’s Balanced Salt Solution (without calcium or magnesium, Sigma, Oakville, ON, Canada) on ice before digestion in 35 mm Petri dishes with 0.3 mg/mL Liberase (Sigma) and 50 U/mL DNase (Sigma) in Dulbecco’s Modified Eagle Medium (Thermo Fisher, Waltham, MA, USA). Wounds were manually homogenized with surgical scissors and incubated for 90 min at 37 °C with regular gentle shaking. Digested samples were then passed through a 70 µm cell strainer to generate single-cell suspensions. Cells were centrifuged (10 min, 300 g, 4 °C), washed, and resuspended in FA3 buffer (PBS, 1 mM EDTA, 10 mM HEPES, 1% FBS, pH 7.4) at 4 °C. For staining, FCγR blocking was first performed with anti-CD16 and anti-CD32 antibodies (BD Biosciences, Warszawa, Poland) for at least 20 min. Cell suspensions were then stained with Zombie VioletTM Fixable Viability Kit (BioLegend, San Diego, CA, USA) for 15 min according to the manufacturer’s instructions, followed by 30 min of incubation with the antibody panel. Antibody details: CD64-AF647 (BD Pharminogen, Clone No. X54-5/7.1, 1:50), CD11b-FITC (BioLegend, Clone No. M1/70), CD3-BV421 (BioLegend, Clone No. 17A2, 1:100), B220-BV421 (BD Biosciences, Clone No. RA3-6B2, 1:100), Ly6C-BV510 (BioLegend, Clone No. HK1.4, 1:100), CD45-PE-Cy7 (BD Pharminogen, San Diego, CA, USA, Clone No. 30-F11, 1:100), and Ly6G-PE (BD Pharminogen, Clone No. 1A8, 1:100). Cell suspensions were then washed and fixed (10% PFA, 30 min, 4 °C), filtered through flow tubes with 40 µm filter caps (Falcon, Corning, NY, USA), and run on the Gallios 561 Analyzer (Beckman Coulter, Brea, CA, USA) within the University of Colorado AMC Cancer Center Flow Cytometry Shared Resource Core Facility. To identify immune cell populations, debris and doublets were first excluded using light scatter, followed by selection for all leukocytes using CD45+ expression. Dead cells and lymphocytes were excluded using Zombie V, B220, and CD3 expression. After gating for CD11b expression, the resultant population was then segregated into monocytes/macrophages (CD64+ Ly6G-) or neutrophils (CD64- Ly6G+). Monocytes/macrophages were then assessed for Ly6C expression to determine the Ly6Clow and Ly6Chi populations (Figures S2 and S3). All cell populations were gated using fluorescence minus one (FMO) controls. Data were analyzed using Kaluza flow analysis software, version 2.1 (Beckman Coulter, Brea, CA, USA).

For the analysis of circulating leukocytes, 200 µL of EDTA-anticoagulated whole blood was incubated with eBioscience™ 1X RBC Lysis Buffer for 10 min at room temperature. Samples were then centrifuged (10 min, 300 g, 4 °C), washed, and resuspended in FA3 buffer and stained using the same protocol and antibody panel used for the wounds.

## Data Availability

The data supporting the conclusions of this article will be made available by the authors on request.

## Supplementary Materials

The following supporting information can be downloaded at https://www.mdpi.com/article/10.3390/ijms26104884/s1.
