# Supplementary material for: Increased ROS and Persistent Pro-Inflammatory Responses in a Diabetic Wound Healing Model (db/db): Implications for Delayed Wound Healing
Source: Int J Mol Sci. 2025 May 20;26(10):4884. doi: 10.3390/ijms26104884 (PMC12112478; doi:10.3390/ijms26104884)
Supplement: Supplementary file 1 [file ijms-26-04884-s001.zip › ijms-3637808-supplementary.pdf]

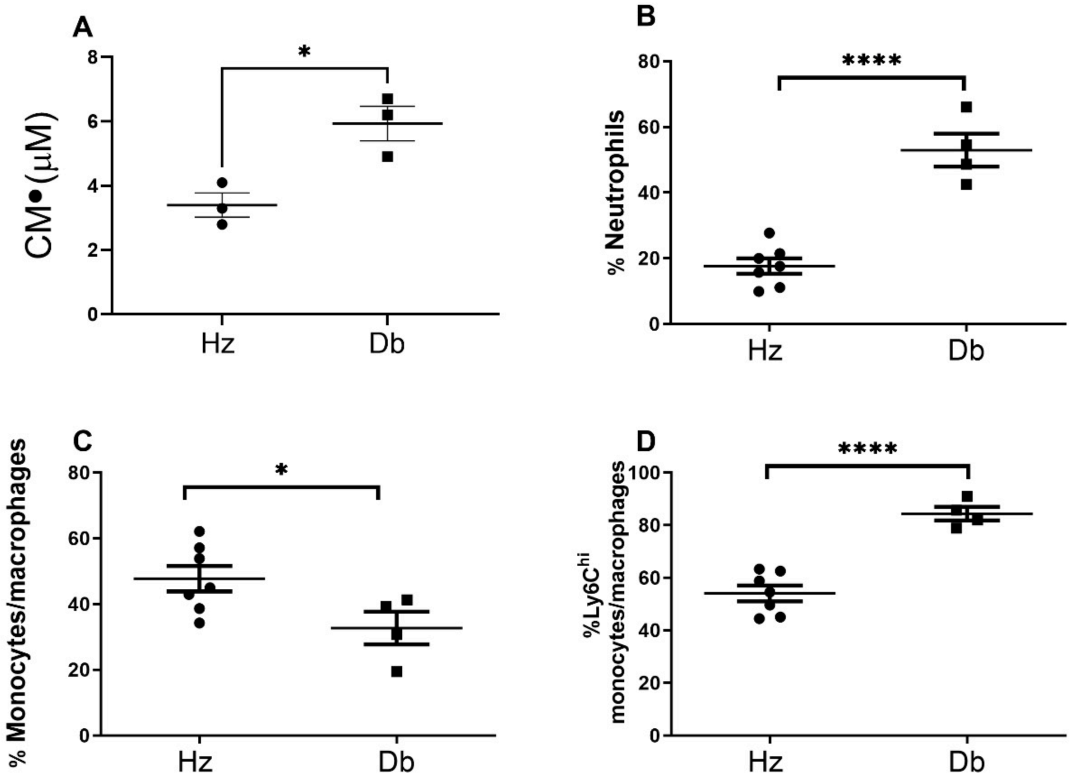

Figure S1. Superoxide level and inflammatory cells count in the blood of diabetic mice compared to heterozygous control mice after wounding. Whole blood was collected via cardiac puncture and fibroblasts were isolated from skin of 12-week-old female control heterozygous (Hz) and diabetic (Db) mice. 300 μl of the blood was treated with CMH probe (0.25mM) for 10 min at room temperature. Cultured fibroblasts treated with CMH probe (0.25μM) incubated 50 min at 5% CO_2 and 37°C. 50 μl of blood or cell suspension were loaded in a capillary tube and EPR measurements done immediately. EPR measurements done at room temperature and spectra were recorded using X-band spectrometer EMXnano (Bruker). The amount of superoxide was determined based on the 1:1 reaction of CMH with superoxide to form CM• and quantified by the SpinFit and Spin Count module (Bruker). (A) Concentration of CM• in blood. Myeloid cell phenotype was assessed by flow cytometry in diabetic and heterozygous blood (B) neutrophils (CD64- Ly6G+), (C) monocytes/macrophages (CD64+ Ly6G-) and (D) monocytes/macrophages Ly6C^{hi} population. Data were analyzed with Prism software using an unpaired t-test and expressed as mean± SEM; *p<0.05 (n=3–7).

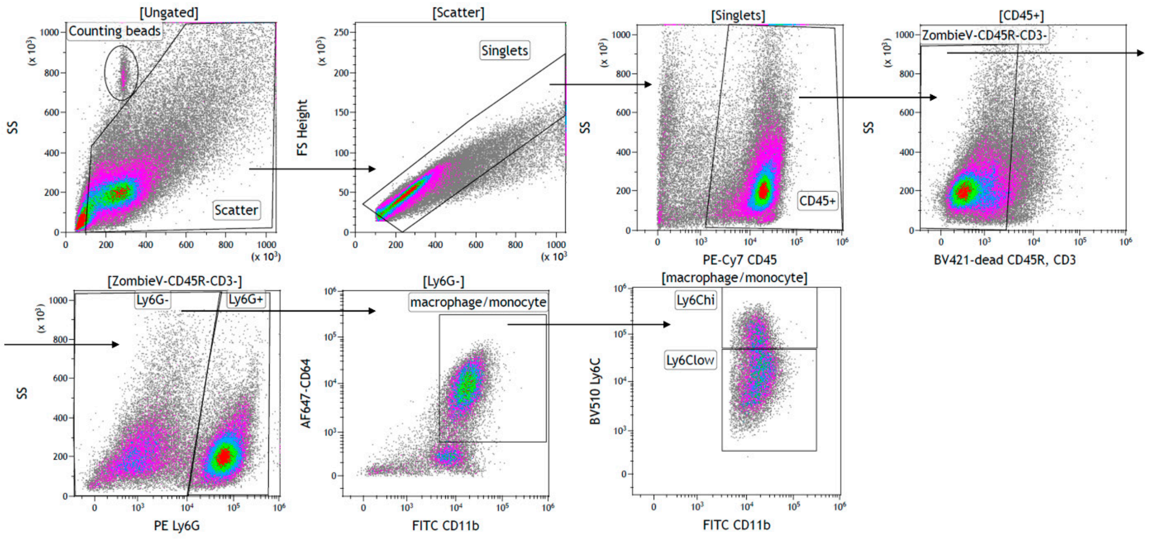

Figure S2. Representative gating strategy (in Hz) for wound samples

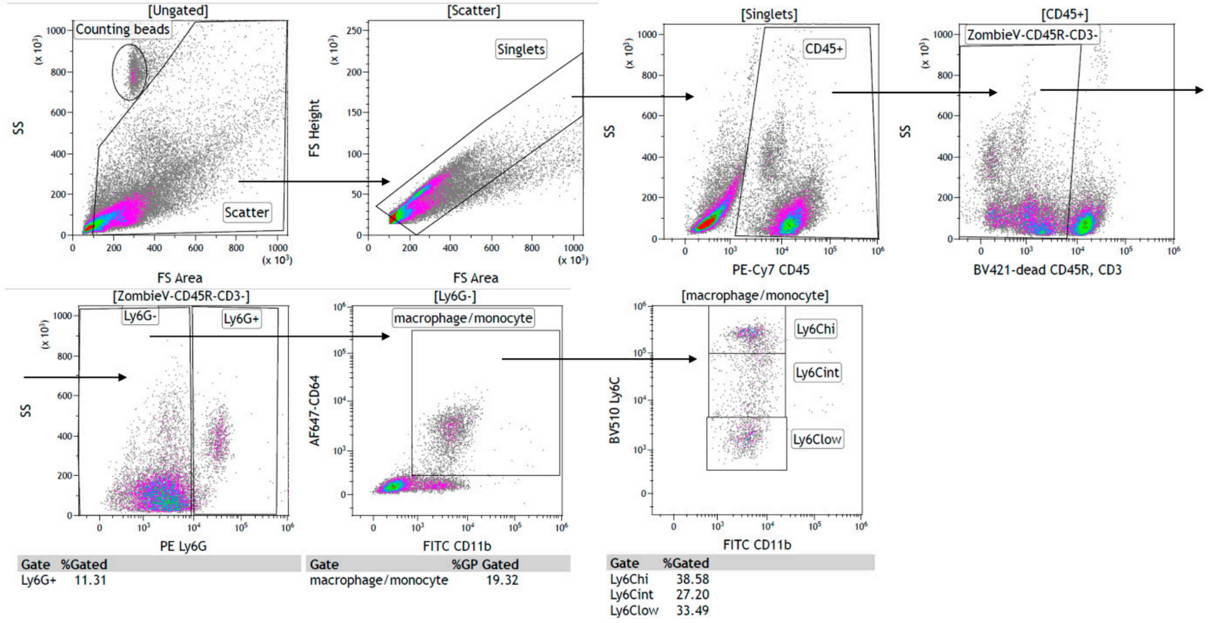

Figure S3. Representative gating strategy (in Hz) for Blood samples
